# A Simple Clinical Measure of Quadriceps Muscle Strength Identifies Responders to Pulmonary Rehabilitation

**DOI:** 10.1155/2014/782702

**Published:** 2014-01-30

**Authors:** James R. Walsh, Norman R. Morris, Zoe J. McKeough, Stephanie T. Yerkovich, Jenny D. Paratz

**Affiliations:** ^1^Queensland Lung Transplant Service, The Prince Charles Hospital, Rode Road, Chermside, QLD 4032, Australia; ^2^School of Medicine, The University of Queensland, St Lucia, QLD 4072, Australia; ^3^Physiotherapy Department, The Prince Charles Hospital, Rode Road, Chermside, QLD 4032, Australia; ^4^School of Rehabilitation Sciences and Griffith Health Institute, Griffith University, Parklands Drive, Southport, QLD 4215, Australia; ^5^Discipline of Physiotherapy, The University of Sydney, 75 East Street, Lidcombe, NSW 2141, Australia

## Abstract

The aim was to determine if baseline measures can predict response to pulmonary rehabilitation in terms of six-minute walk distance (6MWD) or quality of life. Participants with COPD who attended pulmonary rehabilitation between 2010 and 2012 were recruited. Baseline measures evaluated included physical activity, quadriceps strength, comorbidities, inflammatory markers, and self-efficacy. Participants were classified as a responder with improvement in 6MWD (criteria of ≥25 m or ≥2SD) and Chronic Respiratory Questionnaire (CRQ; ≥0.5 points/question). Eighty-five participants with a mean (SD) age of 67(9) years and a mean forced expiratory volume in one second of 55(22)% were studied. Forty-nine and 19 participants were responders when using the 6MWD criteria of ≥25 m and ≥61.9 m, respectively, with forty-four participants improving in CRQ. In a regression model, responders in 6MWD (≥25 m criteria) had lower baseline quadriceps strength (*P* = 0.028) and higher baseline self-efficacy scores (*P* = 0.045). Independent predictors of 6MWD response (≥61.9 m criteria) were participants with metabolic disease (*P* = 0.007) and lower baseline quadriceps strength (*P* = 0.016). Lower baseline CRQ was the only independent predictor of CRQ response. A participant with relatively lower baseline quadriceps strength was the strongest independent predictor of 6MWD response. Metabolic disease may predict 6MWD response, but predictors of CRQ response remain unclear.

## 1. Introduction

Chronic obstructive pulmonary disease (COPD) is a major cause of morbidity and mortality [1]. International statements recommend the implementation of pulmonary rehabilitation for people with COPD who are symptomatic, have decreased functional capacity or reduced health-related quality of life [2]. There is clear evidence that pulmonary rehabilitation can improve exercise capacity, health-related quality of life, and dyspnea [3]. However, whilst the benefits are well documented, not all individuals who meet these criteria are able to access pulmonary rehabilitation [4]. There is also considerable variation in individual program response with between 33% [5] and 53% [6] of participants gaining no meaningful improvement in six minute walk distance (6MWD) and over 50% [5, 6] of participants gaining no meaningful improvement in health-related quality of life. Therefore, a better understanding is required of which participant factors influence program response.

Despite several studies investigating the influence of participant factors on program response, the clinical utility of the findings remains unclear. Lower baseline quadriceps strength [6, 7] or greater quadriceps contractile fatigue [8] possibly best identify responders in exercise capacity. However, the measures used to assess quadriceps muscle strength and fatigue were a Cybex II dynamometer [7], a Cybex Norm Testing and Rehabilitation System [6], and a muscle stimulator [8], respectively. Therefore, it is unclear how the measures used to assess quadriceps muscle strength and fatigue can be incorporated into a routine clinical environment. Hand-held dynamometry is a more clinically accessible measure of quadriceps strength and, importantly, it has been shown to be a valid measure of muscle strength when compared to isokinetic dynamometry [9]. However, it is unknown if quadriceps strength measured via hand-held dynamometry is a factor that predicts response in exercise capacity following pulmonary rehabilitation.

There are other participant factors that have been suggested as inclusion criteria and/or identified as predictors of pulmonary rehabilitation response that require further investigation. For instance, limited physical activity has been recommended as an inclusion criterion for pulmonary rehabilitation [2]. However, the effect of preprogram physical activity level on response to pulmonary rehabilitation is not known. Moderate to severe dyspnea has been proposed previously as a general indicator [2], although, Evans et al. has subsequently reported that individuals can benefit in exercise performance regardless of the dyspnea grade [10]. One study has shown that participants with lower baseline self-efficacy have a better response in exercise capacity following pulmonary rehabilitation [6]. There are also conflicting findings on the influence of comorbidities, with a higher Charlson comorbidity index [11], metabolic syndrome [11], and osteoporosis [12] being associated with poor response in exercise capacity. Identifying a responder in quality of life also appears difficult, with only lower baseline quality of life [7], greater quadriceps contractile fatigue [8], higher 6MWD [6], and comorbidities [11] being identified as independent predictors. Furthermore, the presence of inflammatory markers, including Interleukin-6, Interleukin-8, and C Reactive Protein (CRP), has been associated with reduced quadriceps strength [13] and quality of life [14] in people with COPD and may be able to identify responders to pulmonary rehabilitation.

Therefore, the study's primary aim was to determine if the measures of quadriceps strength, physical activity or inflammatory markers, in combination with dyspnea, comorbidities, and self-efficacy, can predict responders in exercise capacity or quality of life following pulmonary rehabilitation. As the threshold used to define 6MWD appears important in identifying responders [15], a second aim was to evaluate if different methods used to define improvement in 6MWD affected the predictors of response.

## 2. Materials and Methods

This study used a prospective observational cohort design. Inclusion criteria were all participants with stable COPD, who completed baseline assessment at the single institution's pulmonary rehabilitation program between January 2010 and March 2012. Exclusion criteria were participants who did not complete the reassessment measures on program completion or participants who missed ≥ four supervised sessions. Approval was gained from the institutional ethics committees prior to recruitment (HREC/08/QPCH/116-EC28116 and 2009000403) and all participants provided informed consent.

### 2.1. Measurements

Demographic information and medical history were collected at initial assessment. The lung function measures of forced expiratory volume in one second (FEV_1_) and diffusing capacity of the lung for carbon monoxide (DLCO) were assessed prior to program commencement according to standard methods [16]. Baseline dyspnea was assessed on initial assessment using the modified MRC dyspnea scale [17, 18]. Participant's exercise capacity and quality of life were assessed at program commencement and on completion. Exercise capacity was assessed by the 6MWD as per established guidelines [19]. The better of two 6MWD was used at baseline assessment. Quality of life was assessed using the self-reported Chronic Respiratory Questionnaire (CRQ) [20].

The following baseline participant factors were collected.

#### 2.1.1. Quadriceps Strength

Quadriceps strength was measured at initial assessment using hand-held dynamometry (Lafayette Manual Muscle Test System) as per the published protocol [21] with an adjustable strap added. The adjustable strap was secured behind the participant's leg and held to the dynamometer to ensure an isometric contraction. Participants performed three maximal knee extension efforts on each leg with at least one minute rest between tests. In a pilot study, the test-retest reliability for this technique was *r* = 0.996 with a coefficient of variation of 2.7%. In order to normalise this measure, quadriceps strength was expressed as a percentage calculated by adding the best attempt on each leg together (kilograms) and dividing by the participant's body weight.

#### 2.1.2. Physical Activity

Physical activity level was estimated for a cohort of the participants using the SenseWear Pro 3 Armband (SenseWear, Bodymedia Inc.). Participants were asked to wear the armband on the upper right arm for one week from program commencement using standardised protocols [22]. Minimum requirements were for the participant to wear the multisensor device for ≥20 hours/day on a minimum of four days.

#### 2.1.3. Measurement of Inflammatory Markers

Plasma was obtained from peripheral blood samples at initial assessment in participants prior to exercise. The inflammatory markers of Interleukin-6, Interleukin-8, and C Reactive Protein (CRP) were used. Interleukin-6 and Interleukin-8 were measured by in-house ELISA (BD biosciences, lower limit of detection 30 pg/mL). CRP was measured by commercially available ELISA (R&D Systems, USA, lower limit of detection of 0.78 ng/mL).

#### 2.1.4. Comorbidities

The Charlson comorbidity index was calculated at the initial assessment from the participant's medical history [23]. The participant's comorbidities were grouped into the following categories: musculoskeletal, cardiac, and metabolic disease as per the previously described method [11].

#### 2.1.5. Self-Efficacy

Baseline self-efficacy was assessed using the COPD self-efficacy scale at initial assessment [24]. The COPD self-efficacy scale consists of 34 items and it has good test-retest reliability and internal consistency reliability [24]. Self-efficacy was calculated by averaging the total score by the number of questions answered [6].

### 2.2. Pulmonary Rehabilitation Program

The multidisciplinary program was a twice weekly, eight week program. The supervised exercise program included lower limb endurance training and upper and lower limb strength training [25]. The lower limb endurance training consisted of a minimum of twenty minutes of walking and/or cycling per session [25]. The prescribed walking program was commenced at 80% of each participant's average walking speed achieved during the six minute walk test [25]. The cycling program was on a bicycle ergometer with the work rate started at 80% intensity and calculated from the participant's 6MWD [26]. As a guide for training intensity, the participant's walking or cycling intensity was progressed throughout the program to target a four rating or “somewhat severe” of dyspnea or fatigue from the Borg scale [2]. Participants were prescribed a strengthening program of one to three sets/exercise, with the aim of attaining muscle fatigue between six to ten repetitions. The training load was increased when the participant was able to complete ten repetitions. All participants were given a home program and encouraged to complete at least three sessions each week of lower limb endurance and strength training [2].

### 2.3. Defining a Responder

Improvements in exercise capacity and quality of life were used to define a program responder. Due to the debate in defining the important difference in the 6MWD, both the smallest published criteria [27] and the coefficient of repeatability method [15] were used. Therefore, a participant was considered a responder if 6MWD increased by ≥25 m [27] or ≥2SD [15]. For the purpose of this study a responder in CRQ was defined as improvement ≥ 0.5 points/question [20, 28].

### 2.4. Statistical Analysis

Data were analysed using parametric and nonparametric tests as appropriate. Participants were grouped as responders or nonresponders in 6MWD and CRQ. The coefficient of repeatability was calculated from the difference between the baseline 6MWDs. Age, physical activity, dyspnea, quadriceps strength, Interleukin-8, CRP, comorbidities, self-efficacy, baseline assessment 6MWD, and CRQ were examined in a univariate model with significant outcome measures (*P* < 0.1) analysed using a multivariate binary logistic regression model. As there were two different criteria used to define a responder in 6MWD, the Receiver Operating Characteristic ROC curve was used to determine the goodness-of-fit to evaluate the sensitivity (true positive) and specificity (false positive) of the different 6MWD logistic regression models. A sample size of eighty-five participants was needed to detect a 10% difference (power of 0.9 and *P* < 0.05) in multiple factors including quadriceps strength, dyspnea, and self-efficacy score. SPSS version 21 was used to perform the statistical analysis.

## 3. Results

**Figure 1:**
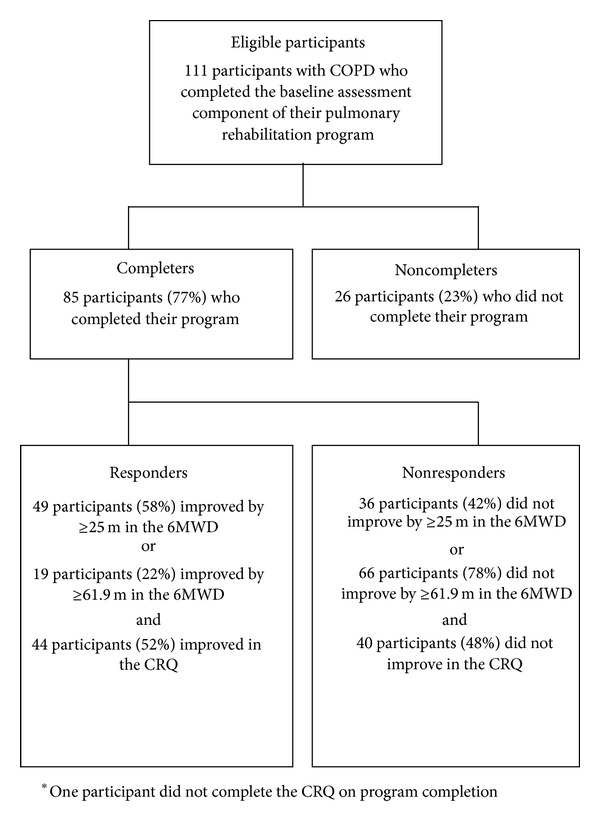
Study flow chart.

Eighty-five eligible participants, with thirty-six females, a mean (±SD) age of 67.4 ± 9.1 years, FEV_1_% of 55.4 ± 22.3%, and DLCO% of 54.2 ± 19.4%, completed pulmonary rehabilitation and were included (Figure 1). Forty-five participants (52.9%) had a baseline mMRC dyspnea grade of ≥2. Sixty-two participants (72.9%) had ≥ one additional comorbidity. Twenty-seven participants (31.8%) were categorised with musculoskeletal disease, twenty-five participants (29.4%) with cardiac disease, and twenty-five participants (29.4%) with metabolic disease. Physical activity level was assessed in forty-six participants. Inflammatory markers were analysed in seventy-seven participants, with all participants having detectable levels of CRP but only four participants had detectable levels of Interleukin-6 and thirty-four participants had detectable levels of Interleukin-8.

Twenty-six participants (26/111) did not complete the program during the study period due to illness (*n* = 15), musculoskeletal injury (*n* = 5), transport difficulties (*n* = 5), and other commitments (*n* = 7), with participants providing multiple reasons for noncompletion. Program completers had a higher mean baseline 6MWD (406 ± 107 m versus 354 ± 133 m; *P* = 0.041) and CRQ (86.7 ± 21.5 versus 73.3 ± 29.3; *P* = 0.011) when compared to noncompleters. Program completers were not significantly different in any other baseline factor (see Table 1 in the Supplementary Material available online at http://dx.doi.org/10.1155/2014/782702).

The mean difference between the two baseline 6MWDs at initial assessment was 18.5 ± 30.9 m (*r* = 0.938) and, therefore, the coefficient of repeatability (2SD) was 61.9 m. Participant's mean overall improvement in 6MWD, from initial assessment to program completion, was 32.6 m (*P* < 0.001). Forty-nine (58%) and nineteen (22%) participants were classified as responders when using the 6MWD criteria of ≥25 m and ≥61.9 m, respectively. The mean improvement in the CRQ was 11.2 points (*P* < 0.001) with forty-four participants (52%) classified as a responder. Twenty-six (59%) and eleven (25%) participants responded in both CRQ and 6MWD when using ≥25 m and ≥61.9 m criteria, respectively.

Participant factors were evaluated for baseline differences in program response. When using the ≥25 m 6MWD criteria, responders were 11.1% lower in mean quadriceps strength (*P* = 0.025) and 0.4 points higher in the self-efficacy score (*P* = 0.025) when compared to nonresponders. Similarly, with the ≥61.9 m criteria, responders were 18.1% lower in mean quadriceps strength (*P* = 0.002) when compared to nonresponders (Table 1). In this model, there was a significant difference in baseline 6MWD between responders (359 ± 20 m) and nonresponders (420 ± 13 m; *P* = 0.015). There was also a relationship between 6MWD response and both musculoskeletal (Fisher's exact test *P* = 0.048) and metabolic disease (*P* = 0.004). No other factor, including physical activity, was significantly different in either 6MWD model. The only significant difference between responders and nonresponders in CRQ was that responders (79 ± 3) had a lower baseline CRQ when compared to nonresponders (95 ± 4; *P* = 0.001). There were no significant differences between responders and nonresponders in any other participant factor (Table 2).

Responders in 6MWD and CRQ were assessed using logistic regression models with responders (Yes = 1 and No = 0) as the dependent variable. Significant participant characteristics (*P* < 0.1) in the univariate models were assessed in a multivariate analysis. Using the 6MWD criteria of ≥25 m, the univariate analysis showed a difference between responders and nonresponders in quadriceps strength and self-efficacy. In the multivariate analysis, lower baseline quadriceps strength (*P* = 0.028) and higher self-efficacy scores (*P* = 0.045) were identified as independent predictors of 6MWD response (Table 3). Using the 6MWD criteria of ≥61.9 m, the univariate analysis indicated a difference between responders and nonresponders in quadriceps strength, baseline 6MWD, and metabolic and musculoskeletal disease. In the multivariate model, lower baseline quadriceps strength (*P* = 0.016) and metabolic disease (*P* = 0.007) were identified as independent predictors. No other participant factor contributed to either 6MWD regression model. Quadriceps strength was the only measure that was an independent predictor of response in both 6MWD models, with these findings translating to a participant 10% weaker in baseline strength increasing the odds of a favourable response in 6MWD by a factor of approximately 1.27 in the ≥25 m model and a factor of approximately 1.51 in the ≥61.9 m model. The Receiver Operating Characteristic curve was used to determine the goodness-of-fit of the 6MWD models. Using the 6MWD criteria of ≥25 m, the area under the curve was 0.608 (95% CI: 0.484–0.733; *P* = 0.092). When using the 6MWD criteria of ≥61.9 m, the area under the curve was 0.803 (95% CI: 0.688–0.917; *P* ≤ 0.001).

The CRQ univariate analysis indicated a difference between responders and nonresponders in the baseline CRQ, the Charlson comorbidity index, and participants with metabolic disease. In the multivariate model, only baseline CRQ (*P* = 0.003) was identified as an independent predictor of response in CRQ (Table 4). No other participant factor contributed to the model.

## 4. Discussion 

The present study investigated a more clinically accessible measure of quadriceps strength and a multisensor device to estimate physical activity level, along with assessing inflammatory markers, dyspnea, comorbidities, and self-efficacy measures to better understand predictors of response following pulmonary rehabilitation. The only predictor that consistently identified response in 6MWD following pulmonary rehabilitation, no matter what model was used to define improvement, was lower baseline quadriceps strength. Higher baseline self-efficacy scores and participants with metabolic disease were independent predictors of response in 6MWD when using ≥25 m and ≥61.9 m models, respectively. In the current study, identifying independent predictors of response in CRQ was difficult with only lower baseline CRQ scores being identified.

Not surprisingly, the method used to define the minimally important difference for 6MWD impacted on the number of participants classified as responders and nonresponders. Although there was a relatively lower number of participants being classified as a responder in 6MWD when using the ≥61.9 m criteria, this model's sensitivity was significantly stronger as demonstrated by a better goodness-of-fit in the Receiver Operating Characteristic curve analysis. Furthermore, the baseline quadriceps strength's mean difference increased between responders and nonresponders and the odds ratio also improved when using the ≥61.9 m model to define 6MWD response. Importantly, lower baseline quadriceps strength was identified as an independent predictor of response regardless of the model used and these findings support the previous results by Troosters et al. (*n* = 49) [7] and Garrod et al. (*n* = 51) [6]. Furthermore, our relatively simple method of using hand-held dynamometry to assess quadriceps strength can be more easily translated into the clinical pulmonary rehabilitation environment which also broadens the applicability of our findings. Metabolic disease was associated with 6MWD response only when using ≥61.9 m criteria to define improvement. There were conflicting results with self-efficacy, as this measure was found to be an independent predictor of 6MWD response using the ≥25 m criteria, but not when using the ≥61.9 m criteria. Our conflicting results would suggest that self-efficacy is not a useful indicator of 6MWD response.

In the current study, baseline physical activity level and dyspnea grade did not identify responders to pulmonary rehabilitation, despite people with these criteria being recommended for program inclusion [2]. Our findings support those by Evans et al. which reported no significant difference in exercise performance between participants with different dyspnea grades [29]. The current study is novel in being the first to assess whether physical activity level could identify responders to pulmonary rehabilitation. While only forty-six participants had this measure assessed due, in part, to the limited availability of the multisensor devices this sample size was sufficient to detect a 10% difference in physical activity during the univariate analyses. Although improving physical activity remains an important goal of pulmonary rehabilitation [30], our findings would suggest that participants can benefit from pulmonary rehabilitation regardless of preprogram physical activity level.

Interleukin-6, Interleukin-8, and CRP also did not identify responders in 6MWD or CRQ in the current study, despite inflammatory markers being previously associated with decreased quadriceps strength [13, 31] and quality of life [14]. Spruit et al. also concluded that markers of systemic inflammation do not adequately identify 6MWD or quality of life response following pulmonary rehabilitation [14]. Although all participants in our study had detectable levels of CRP, ≤44% of participants had detectable levels of Interleukin-6 and Interleukin-8. With persistent systemic inflammation being perhaps more important given the association with mortality [32], an increased rate of COPD exacerbation [32], and an increased risk of comorbidities [33], it may have been more useful to assess participant's inflammatory markers at several time points before, during, and at the end of the pulmonary rehabilitation program. Therefore, due to the possible variation in inflammatory markers over an outpatient pulmonary rehabilitation program, one sample per participant may be inadequate to identify program response.

Similar to our cohort, previous studies have reported a large percentage of pulmonary rehabilitation participants having additional comorbidities [11, 12]. In the current study, metabolic disease was an independent predictor of 6MWD response only when using the ≥61.9 m criteria. This finding supports our previous study [34] but it is contradictory to the findings by Crisafulli et al. which found that metabolic disease was inversely related to 6MWD response [11]. This variation in findings, despite using the same classification method, may be because grouping different diseases into the categories of musculoskeletal and metabolic disease may have masked the ability of these categories to consistently identify pulmonary rehabilitation response. The Charlson comorbidity index was not identified as an independent predictor of response which supports Crisafulli et al. finding [11, 12]. The conflicting findings suggest that further investigation is required to better define the severity of comorbidities to understand the influence on pulmonary rehabilitation response.

The current study also assessed multiple factors with the aim to better understand the influence of participant factors on response in quality of life. However, lower baseline CRQ score was the only independent predictor of response in CRQ. These findings support the previous conclusions by Troosters et al. [7] but are not particularly useful in increasing the understanding of what participant factors identify a responder in CRQ.

Lower baseline quadriceps strength and participants with metabolic disease were identified as independent predictors of response in 6MWD with the threshold used to define improvement in 6MWD an important consideration. Our findings suggest that quadriceps strength becomes a better predictor of response when using a larger threshold to define improvement in 6MWD. In addition, hand-held dynamometry identified participants with weaker baseline strength that were more likely to respond in 6MWD and this result is similar to the previous findings [6, 7]. Importantly, hand-held dynamometry has been shown previously to be a valid measure of muscle strength when compared to isokinetic dynamometry [9] and in our hands has excellent test-retest reliability. However, it is unclear if participants with relatively lower baseline quadriceps strength are more likely to improve in other measures of exercise capacity or important pulmonary rehabilitation objectives such as improving physical activity [30]. Therefore, based upon 6MWD response alone, it is difficult to recommend that participants with relatively lower baseline quadriceps strength should be preferentially considered for pulmonary rehabilitation referral. Further research should investigate whether lower baseline quadriceps strength identifies response to other program objectives such as improving physical activity and if different methods of delivering pulmonary rehabilitation improve the overall number of participants that respond to pulmonary rehabilitation.

## 5. Conclusions 

Quadriceps strength was the strongest independent predictor of response in 6MWD following pulmonary rehabilitation with this measure becoming a better predictor of response when using a larger threshold to define improvement. Metabolic disease may be useful in predicting 6MWD response, but predictors of CRQ response remain unclear.

## Supplementary Material

The supplementary material consists of two data sets of information related to the manuscript entitled A Simple Clinical Measure of Quadriceps Muscle Strength Identifies Responders to Pulmonary Rehabilitation. Supplementary Table 1 consists of participant's baseline assessment data for pulmonary rehabilitation program completers compared to non-completers. Supplementary Table 2 consists of baseline assessment data for participants who wore the multi-sensor device compared to the remaining cohort.

## Figures and Tables

**Table 1:** Baseline assessment data for responders compared to nonresponders in the six minute walk distance.

	(A) Responders	(A) Nonresponders		(B) Responders	(B) Nonresponders	
Number (%)	49 (57.6%)	36 (42.4%)		19 (22.4%)	66 (77.6%)	
Age (years)	68.0 ± 8.6	66.7 ± 9.7	*P* = 0.523	68.3 ± 8.1	67.2 ± 9.5	*P* = 0.628
FEV_1_% predicted	57.0 ± 23.3	53.3 ± 21.3	*P* = 0.458	62.8 ± 26.6	53.2 ± 20.6	*P* = 0.101
mMRC	1.6 ± 1.1	1.7 ± 0.9	*P* = 0.964	1.8 ± 1.1	1.6 ± 1.0	*P* = 0.570
Quadriceps strength (%)	57.5 ± 21.9	68.6 ± 21.9	*P* = 0.025	48.1 ± 16.5	66.2 ± 22.4	*P* = 0.002
Physical activity level (METs)^+^	1.49 ± 0.18	1.54 ± 0.22	*P* = 0.443	1.45 ± 0.15	1.52 ± 0.20	*P* = 0.307
Interleukin-8 (pg/mL)*	151 ± 434	175 ± 485	*P* = 0.818	144 ± 370	167 ± 478	*P* = 0.856
C Reactive Protein (pg/mL)*	7217 ± 13805	10771 ± 10813	*P* = 0.223	9643 ± 18638	8684 ± 10711	*P* = 0.793
COPD self-efficacy score	3.0 ± 0.8	2.6 ± 0.7	*P* = 0.060	2.8 ± 0.5	2.8 ± 0.8	*P* = 0.884
Charlson comorbidity index	1.9 ± 1.1	2.0 ± 1.1	*P* = 0.608	2.3 ± 1.1	1.8 ± 1.1	*P* = 0.126
Metabolic disease	15/49 (31%)	10/36 (28%)	*P* = 0.814	11/19 (31%)	14/66 (28%)	*P* = 0.004
Cardiac disease	13/49 (27%)	12/36 (33%)	*P* = 0.631	5/19 (26%)	20/66 (30%)	*P* = 1.000
Musculoskeletal disease	18/49 (37%)	9/36 (25%)	*P* = 0.346	10/19 (31%)	17/66 (28%)	*P* = 0.048
Baseline 6MWD (m)	393 ± 16	423 ± 17	*P* = 0.202	359 ± 20	420 ± 13	*P* = 0.015

Part (A) responders/non-responders used the criteria of six-minute walk distance (6MWD) ≥25 m. Part (B) responders/non-responders used the criteria of 6MWD ≥61.9 m. Data expressed as the mean ± standard deviation. FEV_1_: forced expiratory volume in one second, mMRC: modified Medical Research Council dyspnea scale, and METs: metabolic equivalent of task.

*Systemic inflammatory markers were assessed in 77 participants.

^+^Physical activity level was assessed in 46 participants.

**Table 2:** Baseline assessment data for responders compared to non-responders in the Chronic Respiratory Questionnaire.

	Responders	Nonresponders	
Number (%)	44 (52.3%)	40 (47.6%)	
Age (years)	66.6 ± 8.2	68.3 ± 10.3	*P* = 0.385
FEV_1_% predicted	55.5 ± 23.0	55.0 ± 22.1	*P* = 0.925
mMRC	1.8 ± 1.1	1.6 ± 1.0	*P* = 0.384
Quadriceps strength (%)	64.8 ± 23.1	59.4 ± 22.0	*P* = 0.278
Physical activity level (METs)^+^	1.52 ± 0.25	1.49 ± 0.20	*P* = 0.697
Interleukin-8 (pg/mL)*	201 ± 514	120 ± 384	*P* = 0.438
C Reactive Protein (pg/mL)*	8765 ± 11327	8998 ± 14108	*P* = 0.937
COPD self-efficacy score	2.8 ± 0.7	2.9 ± 0.8	*P* = 0.554
Charlson comorbidity index	2.2 ± 1.2	1.7 ± 0.8	*P* = 0.051
Metabolic disease	17/44 (39%)	8/40 (20%)	*P* = 0.062
Cardiac disease	12/44 (27%)	13/40 (33%)	*P* = 0.639
Musculoskeletal disease	15/44 (34%)	12/40 (30%)	*P* = 0.688
Baseline CRQ	79 ± 3	95 ± 4	*P* = 0.001

Data expressed as the mean ± standard deviation. FEV_1_%: forced expiratory volume in one second, mMRC: modified Medical Research Council dyspnea scale, METs: metabolic equivalent of task, and CRQ: Chronic Respiratory Questionnaire.

*Systemic inflammatory markers were assessed in 77 participants.

^+^Physical activity level was assessed in 46 participants.

**Table:** (a) Binary logistic regression analysis—responders defined as ≥25 m increase in 6MWD

	*β*	SE	Wald *χ* ^2^	*P *	Odds ratio (Exp *β*)	95% CI for Exp *β*
Univariate analysis						
Quadriceps strength	−0.023	0.011	4.737	0.030	0.977	0.957–0.998
COPD self-efficacy score	0.565	0.304	3.450	0.063	1.760	0.969–3.196
Multivariate analysis						
Quadriceps strength	−0.024	0.011	4.822	0.028	0.976	0.955–0.997
COPD self-efficacy score	0.626	0.313	4.015	0.045	1.871	1.014–3.451

**Table:** (b) Binary logistic regression analysis—responders defined as ≥61.9 m increase in 6MWD

	*β*	SE	Wald *χ* ^2^	*P *	Odds ratio (Exp *β*)	95% CI for Exp *β*
Univariate analysis						
Quadriceps strength	−0.048	0.017	8.291	0.004	0.953	0.922–0.985
Metabolic disease	−1.631	0.554	8.673	0.003	0.196	0.066–0.580
Musculoskeletal disease	−1.164	0.539	4.666	0.031	0.312	0.109–0.898
Baseline 6MWD	−0.006	0.003	4.592	0.032	0.994	0.989–1.000
Multivariate analysis						
Quadriceps strength	−0.043	0.018	5.776	0.016	0.958	0.924–0.992
Metabolic disease	−1.762	0.648	7.391	0.007	0.172	0.048–0.612
Baseline 6MWD	−0.006	0.003	3.008	0.083	0.994	0.988–1.001

Only variables with *P* < 0.1 are shown in the table.

**Table 4:** Binary logistic regression model for a responder in the Chronic Respiratory Questionnaire (CRQ).

	*β*	SE	Wald *χ* ^2^	*P *	Odds ratio (Exp *β*)	95% CI for Exp *β*
Univariate analysis						
Charlson comorbidity index	0.426	0.223	3.643	0.056	1.531	0.989–2.370
Metabolic disease	−0.924	0.502	3.384	0.066	2.519	0.941–6.738
Baseline CRQ	−0.038	0.012	9.486	0.002	0.962	0.939–0.986
Multivariate analysis						
Metabolic disease	−0.914	0.539	2.872	0.090	0.401	0.139–1.154
Baseline CRQ	−0.038	0.013	9.100	0.003	0.963	0.939–0.987

Only variables with *P* < 0.1 are shown in the table.

## Abbreviations

COPD: Chronic obstructive pulmonary disease
CRP: C Reactive Protein
CRQ: Chronic Respiratory Questionnaire
DLCO: Diffusing capacity of the lung for carbon monoxide
FEV_1_: Forced expiratory volume in one second
mMRC: modified Medical Research Council dyspnea scale
6MWD: Six-minute walk distance.
