# Supplementary material for: A Simple Clinical Measure of Quadriceps Muscle Strength Identifies Responders to Pulmonary Rehabilitation
Source: Pulm Med. 2014 Jan 30;2014:782702. doi: 10.1155/2014/782702 (PMC3929516; doi:10.1155/2014/782702)
Supplement: Supplementary file 1 — The supplementary material consists of two data sets of information related to the manuscript entitled A Simple Clinical Measure of Quadriceps Muscle Strength Identifies Responders to Pulmonary Rehabilitation. Supplementary Table 1 consists of participant's baseline assessment data for pulmonary rehabilitation program completers compared to non-completers. Supplementary Table 2 consists of baseline assessment data for participants who wore the multi-sensor device compared to the remaining cohort. [file 782702.f1.docx]

Supplementary Table 1: Participant's baseline assessment data for completers compared to non-completers.

|  | Completers | Non-completers |  |
| --- | --- | --- | --- |
| Number (%) | 85 | 26 |  |
| Age (years) | 67.4 ± 9.1 | 67.2 ± 9.5 | *p*=0.930 |
| FEV_1_% predicted | 55.4 ± 22.4 | 51.4 ± 22.5 | *p*=0.452 |
| mMRC | 1.7 ± 1.0 | 2.0 ± 1.0 | *p*=0.111 |
| Quadriceps strength (%) | 62.3 ± 22.5 | 60.1 ± 21.8 | *p*=0.681 |
| Interleukin-8 (pg/ml)+ | 161.6 ± 454.3 | 12.6 ± 20.4 | *p*=0.305 |
| C Reactive Protein (pg/ml)* | 9637 ± 17829 | 9600 ± 9454 | *p*=0.995 |
| COPD self-efficacy score (mean score/question) | 2.8 ± 0.8 | 2.8 ± 0.8 | *p*=0.924 |
| Charlson Co-morbidity index | 1.9 ± 1.1 | 1.9 ± 1.0 | *p*=0.851 |
| Baseline 6MWD (m) | 406 ± 107 | 350 ± 134 | *p*=0.030 |
| Baseline CRQ | 86.7 ± 21.5 | 73.2 ± 29.9 | *p*=0.012 |

Categorical data expressed as a ratio (%). Continuous data expressed as the mean ± standard deviation. FEV_1_ = forced expiratory volume in one second, mMRC = modified Medical Research Council dyspnea scale, 6MWD = six minute walk distance, CRQ = Chronic Respiratory Questionnaire.

* 10 participants classified as a non-completer provided a sample for C Reactive Protein comparison.

+ Only 3 participants (3/10) classified as a non-completer had detectable levels of Interleukin-8 compared to 34 participants in the completers group.
