# Supplementary material for: A Simple Clinical Measure of Quadriceps Muscle Strength Identifies Responders to Pulmonary Rehabilitation
Source: Pulm Med. 2014 Jan 30;2014:782702. doi: 10.1155/2014/782702 (PMC3929516; doi:10.1155/2014/782702)
Supplement: Supplementary file 2 [file 782702.f2.docx]

Supplementary Table 2: Baseline assessment data for participants who wore the multi-sensor device compared to the remaining cohort.

|  | Participants who wore device | Participants who didn't wear device |  |
| --- | --- | --- | --- |
| Number (%) | 46 (54.1%) | 39 (45.9%) |  |
| Age (years) | 67.3 ± 8.8 | 67.6 ± 9.7 | *p*=0.889 |
| FEV_1_% predicted | 54.8 ± 23.1 | 56.1 ± 21.7 | *p*=0.801 |
| Body mass index (kg.m^-2^) | 27.9 ± 5.2 | 27.3 ± 5.3 | *p*=0.646 |
| Quadriceps strength (%) | 60.8 ± 20.7 | 64.2 ± 24.6 | *p*=0.496 |
| COPD self-efficacy score | 2.9 ± 0.9 | 2.7 ± 0.7 | *p*=0.341 |
| Charlson Co-morbidity index | 1.9 ± 1.2 | 2.0 ± 1.0 | *p*=0.880 |
| Six minute walk distance (m) | 415 ± 110 | 396 ± 104 | *p*=0.410 |
| CRQ | 88.1 ± 22.7 | 85.1 ± 20.1 | *p*=0.513 |

Data expressed as the mean ± standard deviation. FEV_1_% = forced expiratory volume in one second, CRQ = Chronic Respiratory Questionnaire.
